# The Impact of COVID-19 Pandemic on the Learning Outcomes of Medical Students in Taiwan: A Two-Year Prospective Cohort Study of OSCE Performance

**DOI:** 10.3390/ijerph19010208

**Published:** 2021-12-25

**Authors:** Tzyy-Yurn Tzeng, Chia-An Hsu, Ying-Ying Yang, Eunice J. Yuan, Ya-Ting Chang, Tzu-Hao Li, Chung-Pin Li, Jen-Feng Liang, Jiing-Feng Lirng, Tzeng-Ji Chen, Chia-Chang Huang, Ming-Chih Hou, Chen-Huan Chen, Wayne Huey-Herng Sheu

**Affiliations:** 1 Department of Medical Education, Medical Innovation and Research Office, Taipei Veterans General Hospital, Taipei 11217, Taiwan; beckytu31619@gmail.com (T.-Y.T.); cpli@vghtpe.gov.tw (C.-P.L.); jfliang@vghtpe.gov.tw (J.-F.L.); ccchuang7@vghtpe.gov.tw (C.-C.H.); chencc101@gmail.com (C.-H.C.); 2 College of Medicine, National Yang Ming Chiao Tung University, Taipei 11217, Taiwan; nfwya0811@gmail.com (C.-A.H.); eunicejuan@gmail.com (E.J.Y.); laikaislong@gmail.com (Y.-T.C.); pearharry@yahoo.com.tw (T.-H.L.); jflirng@vghtpe.gov.tw (J.-F.L.); tjchen@vghtpe.gov.tw (T.-J.C.); mchou@vghtpe.gov.tw (M.-C.H.); whhsheu@vghtpe.gov.tw (W.H.-H.S.); 3 Department of Ophthalmology, Taipei Veterans General Hospital, Taipei 11217, Taiwan; 4 Clinical Innovation Center, Taipei Veterans General Hospital, Taipei 11217, Taiwan; 5 Division of Allergy, Immunology, and Rheumatology, Department of Internal Medicine, Shin Kong Wu Ho Su Memorial Hospital, Taipei 11101, Taiwan; 6 Department of Internal Medicine, Taipei Veterans General Hospital, Taipei 11217, Taiwan

**Keywords:** medical students, COVID-19, supplemental teaching, OSCE (objective structured clinical examination), standardized patient

## Abstract

Background/Aims: To avoid the negative impacts of the COVID-19 pandemic on clinical clerkship, supplemental teachings such as digital materials in the scenario-based distal simulations were implemented. This study utilized the OSCE (objective-structured clinical examination) to evaluate the impact of COVID-19 pandemic on the learning outcome of medical students from the regular group (class of 2020) and pandemic-impacted group (class of 2021). Methods: All medical students serially took, firstly, the mock-OSCE, secondly, the mock-OSCE, and the national OSCE. Then, the serial OSCE scores were compared between groups. Results: Although with similar scores in the first mock OSCE, the regular group (*n* = 78) had a higher average score in the national OSCE than the pandemic-impacted group (*n* = 80) (872.18 vs. 834.96, *p* = 0.003). In terms of improvement, the performances of the regular group were also better than the pandemic-impacted group between the second mock OSCE and the national OSCE (79.10 vs. 38.14, *p* = 0.014), and between the second mock OSCE and the national OSCE (125.11 vs. 77.52, *p* = 0.003). While separating distinct genres, the regular group had more of a score increment in standardized patient-based stations between the second mock OSCE and the national OSCE (regular vs. pandemic-impacted: 57.03 vs. 18.95, *p* = 0.003), as well as between the first mock OSCE and the national OSCE (75.97 vs. 26.36, *p* < 0.001), but there was no significant difference among the skill-based stations. In particular, the scores of the emergency medicine associated station in the national OSCE of the pandemic-impacted group was lower. Conclusions: Our study implies that the pandemic significantly hampered the learning outcomes of final year medical students in their clinical participation. Especially facing the COVID-19 pandemic, more supplemental teachings are needed to compensate the decreasing emergency medicine exposure.

## 1. Introduction

Coronavirus disease 2019 (COVID-19) was first reported in December 2019 and spread rapidly with an outbreak throughout the whole world, creating more than 217 million cases confirmed and over 4.55 million deaths worldwide as far [1]. Alongside daily life, the pandemic also affected medical education worldwide [2], since the opportunities of patient care and bedside teaching for medical students were restricted in attempts to lower viral transmission and to keep social distances [3]. For students’ safety during the pandemic, some training through clinical rotations were suspended [4], thus a lot of effort has been exerted to ensure the integrity and continuity of medical education, such as pre-recorded lectures, online classes and meetings, as well as virtual, augmented, mixed reality-based materials in scenario-based simulations [5,6].

The medical curriculum in Taiwan is a six-year program, with clinical participation in the last 2 years. Students receive in-hospital rotations during that time. In clerkships, students are assigned to various departments, including internal medicine, surgical medicine, pediatrics, gynecology, and emergency medicine. They acquire clinical knowledge by shadowing and assisting residential physicians in daily practice, primary patient care, bedside procedures, daily record writing, and case presentations. Before the end of the clerkship, all students must take the national objective structured clinical examination (OSCE), which is a necessary process to obtain a medical license. Before national OSCE, medical students will attend formative mock OSCEs to practice and strengthen their clinical skills.

Since the first case of COVID-19 in our country appeared on 21 January 2020, teaching hospitals had developed some strategies in response to the pandemic. First, social distancing was implemented by seating arrangements in most indoor spaces, and some events were cancelled to avoid large crowds. Second, medical students were asked to keep track of their health conditions. Upon entering hospitals, medical students had their temperatures taken and hands sanitized with alcohol. Third, with the establishment of online learning platforms, supplemental teaching was arranged to combine with regular face-to-face teaching [7]. Unfortunately, there became more restrictions on clerkships for medical students after the cluster infection of May 2021. For years, our institution had implemented medical simulation to enhance clinical competence of medical students as well as to improve care quality [8,9]. Parts of patient care demonstrations were replaced by digital clinical modules and the decrease in the rotations in high-risk departments such as the emergency room were compensated by distant real-patient teaching and simulation training.

In our country, faculties were widely trained for designing digital clinical placement sessions [10,11]. It was reported that, although technology played an important role in the efforts to allow the medical curriculum to be delivered, online education still had its weakness, especially because of the need for hands-on learning experiences in medical education. Medical studies had also pointed out that only a few students agreed that online learning could be used for clinical aspects [12].

Collectively, the COVID-19 pandemic has impacted the completeness of medical training in all respects. This study aims to evaluate the impact of the COVID-19 pandemic and supplemental teaching on the learning outcome of medical students by utilizing serial OSCEs.

## 2. Method

### 2.1. Study Design and Data Collection

This prospective observational study was conducted in our hospital, a teaching hospital and medical center in north Taiwan [13]. Medical students in Taiwan participate in mock and national OSCE in their final year, and the medical center holds two sessions of mock OSCE every year prior to medical students taking the national OSCE. Since the COVID-19 pandemic occurred, supplemental teaching such as online lectures was used in the training of medical students, and parts of patient care demonstrations were also trained by virtual education, and the decrease in exposure to high-risk departments such as the emergency room were supplemented with simulation trainings in clerkships. To evaluate the impact of decreased clinical exposure during the COVID-19 pandemic on medical students’ clinical performance, we collected the data of two mock OSCEs and a national OSCE of medical students from a regular group (class of 2020) and a pandemic-impacted group (class of 2021). Medical students from the regular group received the first mock OSCE in September 2019, second mock OSCE in February 2020, and the national OSCE in April 2020; whereas medical students from the pandemic-impacted group received the first mock OSCE in August 2020, second mock OSCE in January 2021, and the national OSCE in April 2021.

### 2.2. The Components and Scoring of OSCEs

OSCE has been put into practice in our country for more than ten years [9,14]. The OSCEs include topics on medicine, surgery, pediatric, obstetrics and gynecology with standardized patient-based or skills-based stations. The first and second mock OSCEs were composed of two parallel tracks of 6 stations (4 standardized patient-based stations and 2 skill-based stations) and medical students rotated either track randomly. The national OSCE included a single track of 12 stations (8 standardized patient-based stations and 4 skill-based stations). Skills-based exams took insight from medical students’ procedural skills, and standardized patients-based exams cover competencies of history taking, physical examination, differential diagnosis, clinical reasoning, and communication skills. It is noted that the mock OSCE is a formative assessment, and no overall pass or fail is provided to the medical students. In each station, faculty raters evaluated medical students’ performance based on a standard checklist [15], giving a performance grade and an initial score in each station. The performance grade could be divided into 5 levels, which include 1 (poor), 2 (fair), 3 (average), 4 (good), and 5 (excellent). Additionally, to ensure equal weighting of all OSCE stations, the initial scores were transformed onto a similar 100-percentage scale. The passing standard of each station was the mean score from the checklist in those rated performance grade 2 in the global rating [16]. All faculty raters and standardized patients attended serial training sessions that included extensive instructions in how to use the checklist and performance grade in practice rating sessions.

### 2.3. Outcome Measurement of OSCEs

Firstly, those with the performance grade 1 or 2 were defined as below the standard, and those with the grade 3 to 5 as above the standard. We then calculated each medical student’s number of stations that was classified in the group above the standard in serial OSCEs for further analysis. Moreover, initial scores were collected and properly adjusted since there was a difference in the number of stations between two mock OSCEs and national OSCE and were then set as another outcome measurement. Score increments were also used to represent students’ improvement in serial OSCEs.

### 2.4. Statistical Analysis

All data collected from mock and national OSCEs were recorded to Microsoft Excel (version 2016, Microsoft Corporation, Redmond, WA 98052-7329, USA) electronically. Data of the baseline characteristics were reported as means and standard deviations. An independent sample *t* test was used for comparisons of performance in each OSCE between medical students from the regular group (class of 2020) and the pandemic-impacted group (class of 2021). It was also used to compare the score improvement of students from two groups in a series of OSCEs. For all analyses, the results were considered significant when *p* < 0.05. All statistical analyses were conducted with IBM SPSS (version 25.0, Armonk, NY, USA, IBM Corp).

### 2.5. Ethical Review

The conduct of the study was approved by Institutional Review Board of the medical center ref: 2018-01-006CC. Consent was exempted for this minimal risk research.

## 3. Results

### 3.1. Baseline Characteristics

Seventy-eight medical students from the regular group (class of 2020) and eighty medical students from the pandemic-impacted group who completed all three examinations were included in the final analysis. While classifying two groups of students by gender, there was no significant difference in the ratio of male-to-female students (proportion of males in the regular group vs. the pandemic-impacted group: 66.67% vs. 65%) and the age distribution of students (regular group vs. pandemic-impacted group: 25.07 vs. 25.14) between the two groups. Comparing the performance (the number of stations with performance above standard) between students from the two groups, no significant difference was noted in the first mock OSCE (regular vs. pandemic-impacted: 4.35 vs. 4.74) and the second mock OSCE (regular vs. pandemic-impacted: 4.47 vs. 4.69), but we could see that those from the regular group had more stations reaching standard than those from the pandemic-impacted group in the national OSCE (regular vs. pandemic-impacted: 11.05 vs. 10.38, *p* = 0.002). Furthermore, there was a similar trend as comparing total scores between two groups, showing no significant difference in the first mock OSCE (regular vs. pandemic-impacted: 747.07 vs. 757.44) and the second mock OSCE (regular vs. pandemic-impacted: 793.08 vs. 796.83), but the regular group had higher total scores than the pandemic-impacted group in the national OSCE (regular vs. pandemic-impacted: 872.18 vs. 834.96, *p* = 0.003) (Table 1).

### 3.2. Comparison of Medical Students’ Improvement between Serial OSCEs

**Figure 1:**
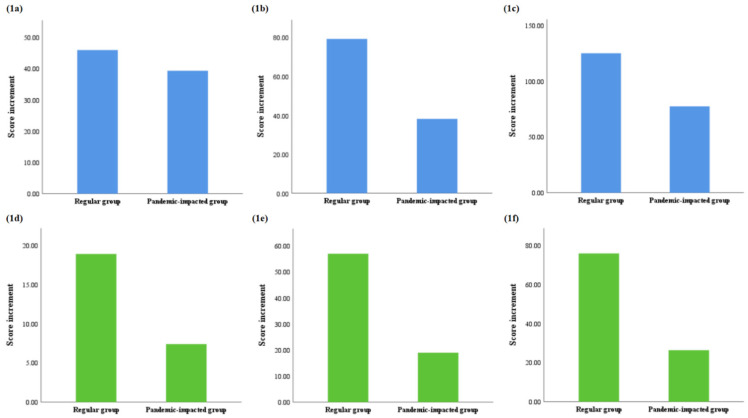
Total score increment of OSCE stations. (**1a**) Total score increment between first mock OSCE and second mock OSCE, *p* = 0.715; (**1b**) total score increment between second mock OSCE and national OSCE, *p* = 0.014; (**1c**) total score increment between first mock OSCE and national OSCE, *p* = 0.003; (**1d**) score increment of standardized patient-based stations between first mock OSCE and second mock OSCE, *p* = 0.404; (**1e**) score increment of standardized patient-based stations between second mock OSCE and national OSCE, *p* = 0.003; (**1f**) score increment of standardized patient-based stations between first mock OSCE and national OSCE, *p* < 0.001. Results were considered significant by *p* < 0.05.

There was no significance difference in the total score increment from the first mock OSCE to the second mock OSCE between the two groups (regular vs. pandemic-impacted: 46.01 vs. 39.38), but the regular group had a greater total score increment from the second mock OSCE to the national OSCE (regular vs. pandemic-impacted: 79.10 vs. 38.14, *p* = 0.014) and from the first mock OSCE to the national OSCE (regular vs. pandemic-impacted: 125.11 vs. 77.52, *p* = 0.003) (Figure 1). Similar results could be noted in the aspect of the score increment in standardized patient-based stations, as the two groups had close improvement from the first mock OSCE to the second mock OSCE (regular vs. pandemic-impacted: 18.94 vs. 7.41), but the regular group had a greater score increment than the pandemic-impacted group in standardized patient-based stations between the second mock OSCE and the national OSCE (regular vs. pandemic-impacted: 57.03 vs. 18.95, *p* = 0.003), and between the first mock OSCE and the national OSCE (regular vs. pandemic-impacted: 75.97 vs. 26.36, *p* < 0.001) (Figure 1).

**Figure 2:**
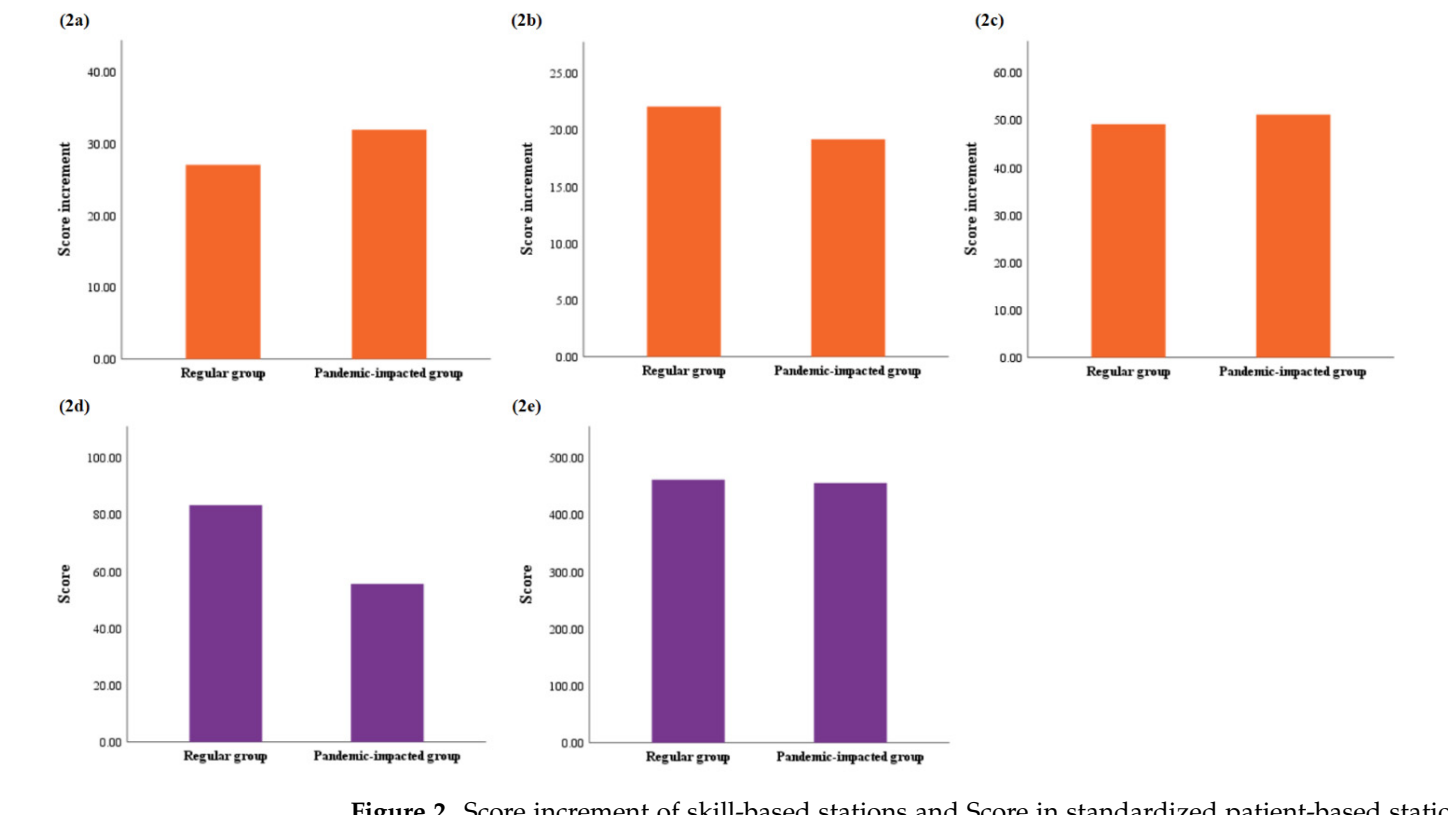
Score increment of skill-based stations and Score in standardized patient-based station. (**2a**) Score increment of skill-based stations between first mock OSCE and second mock OSCE, *p* = 0.564; (**2b**) Score increment of skill-based stations between second mock OSCE and national OSCE, *p* = 0.706; (**2c**) Score increment of skill-based stations between first mock OSCE and national OSCE, *p* = 0.781; (**2d**) National OSCE score comparison of standardized patient-based station associated with emergency medicine, *p* < 0.001; (**2e**) National OSCE score comparison of standardized patient-based station not associated with emergency medicine, *p* = 0.477; Results were considered significant by *p* < 0.05.

However, there was no significant difference between the two groups in terms of the improvement in skill-based stations, as the results show below, from the first mock OSCE to the second mock OSCE (regular vs. pandemic-impacted: 27.07 vs. 31.89), from the second mock OSCE to the national OSCE (regular vs. pandemic-impacted: 22.07 vs. 19.19), and from the first mock OSCE to the national OSCE (regular vs. pandemic-impacted: 49.14 vs. 51.16) (Figure 2).

### 3.3. Comparison of Medical Student’s Performance in Standardized Patient-Based Stations

While focusing on the students’ performance of standardized patient-based stations in the national OSCE, we could further divide the stations into two groups, which were emergency medicine associated and non-emergency medicine associated stations. In non-emergency medicine associated stations, students from the two groups had close scores (regular vs. pandemic-impacted: 461.80 vs. 455.92). On the other hand, the regular group had a higher score than the pandemic-impacted group in the emergency medicine associated station (regular vs. pandemic-impacted: 83.35 vs. 55.64, *p* < 0.001) (Figure 2).

### 3.4. Comparison of Male and Female Students’ Performance and Improvement

We also compared students’ score improvement from the first mock OSCE to the national OSCE by gender, with the result showing no difference in both the regular group (male vs. female: 126.55 vs. 122.22) and the tpandemic-impacted group (male vs. female: 93.02 vs. 50.26). There was also no difference when it came to score increment in standardized patient-based stations and skill-based stations (Table 2).

## 4. Discussion

The results of the study demonstrated that medical students from the regular group had better performance in the national OSCE than those from the pandemic-impacted group, especially in the emergency medicine associated stations. Students from the regular group also had more improvement from mock OSCEs to the national OSCE.

In response to the extensive outbreak of COVID-19 throughout the world, supplemental teaching with various online videos and virtual lectures were wildly used for education in different fields, as well as medical education. Looking back to previous research, there are no significant differences in student performance among delivery modes [17,18]. Similar conclusions were demonstrated that no negative impact of performance when students faced remote teaching in the aspects of business, English, computer programming and communication disciplines [19]. However, when it comes to medical education, there are different opinions [20]. Several studies had pointed out that there still are lots of challenges to train clinical skills that only real-world practice can provide, such as approaching the patients during clinical examination and surgery [21]. The strength of the present study is that we used OSCEs as the outcome measurements of the clinical competence of medical students, which is relatively objective and has been integrated into the national medical examination in our country [22]. The present study also analyzed the performance improvement in serial OSCEs, providing a more comprehensive viewpoint for the impact of the decreased exposure to clinical issues due to the outbreak of COVID-19 and the effects of supplemental teaching.

According to the findings of serial OSCEs in our study, medical students’ clinical learning was still negatively affected by the pandemic despite digital clinical placement having been implemented to maintain the completeness of clerkship. To serve as a reference, we retrieved the data of the previous year (the students from the class of 2019). We could see that the passing rate of the previous year and the regular group were both 100%, and was 93.75% in students from the pandemic-impacted group. The score range in each year was listed below, respectively, as 715.46 to 992.94 in the previous year, from 728.87 to 994.28 in the regular group, and from 649.45 to 982.76 in the pandemic-impacted group. This indicated that students from the regular group and the previous year had similar performances, which were better than the pandemic-impacted group. While discussing the score increment in serial OSCEs in our study, there was no significant difference from the first mock OSCE to the second mock OSCE between the two groups. Nonetheless, the regular group (class of 2020) had a greater improvement from the second mock OSCE to the national OSCE. The possible explanation was that between the first mock OSCE and the second mock OSCE for the pandemic-impacted group (class of 2021), that is, between August 2020 to January 2021, the COVID-19 pandemic situation in our country was relatively stable. The stabilization of the pandemic came from having the international support and hard work of the government to implement specific approaches for case identification, containment, and resource allocation to protect the public health [23]. With the pandemic under control, practical clinical trainings were less affected, and medical students could keep their learning on patient care. Unfortunately, after the cluster infection occurred in north Taiwan in January 2021, not only did people reduce the needs of non-emergency medical treatments [24], hospitals also began to lower the number of beds as some studies of our country reviewed that the number of visits to the outpatient department decreased [25] and the bed occupancy rate of hospitals were reduced [26]. This led to the decrease in medical students’ exposure to patients, and consequently trainees were lacking enough opportunities to practice history taking, physical examinations, and medical communications.

While further analyzing the different categories of the OSCE stations, we could see that the standardized patient-based stations were affected to a greater degree than skill-based stations since the regular group (class of 2020) had a greater score increment than the pandemic-impacted group (class 2021) in standardized patient-based stations between the second mock OSCE and the national OSCE, but there was no significant difference between regular and pandemic-impacted groups in terms of improvement in skill-based OSCE stations. For these findings, it is possible that all procedures in the skills-based stations, such as arterial blood gas puncture, foley catheter placement, and tracheal intubation, were assessed with mannequins either in the mock or national OSCEs. Therefore, before formal assessments, although medical students had less contact with real patients, they still could practice procedural skills repeatedly on mannequins. However, when talking about soft skills, such as history taking, physical examinations, and medical communications, that require practical clinical experience, the pandemic-impacted clerkships did have negative effects on medical students, as the performance of students from the class of 2021 in standardized patient-based stations showed as poor compared to the regular group (class of 2020). In particular, among the standardized patient-based stations, the OSCE performance of the two classes of medical students differed the most in stations that related to emergency medicine, which was one of the course of clerkship that most affected by the COVID-19 pandemic [27].

Facing the COVID-19 pandemic, supplemental teachings were arranged to compensate for the decrease in clinical exposure by the protection of medical students from rotations, especially in emergency medicine. Several previous studies had proposed methods to tackle the challenges, such as the use of telemedicine and digital clinic placement. Telemedicine allows medical students to benefit from it as acquiring clinical experience by interacting with real patients under the supervision of attending physicians [28]; while the digital clinic works the way that students initially receive online cases, including the patient’s history and findings from physical examination, and then they are requested to propose investigation results and management plans [29]. A recent study revealed that students provided overwhelmingly positive feedback to the virtual clinical experiences in emergency medicine clerkships as they appreciated the breadth of chief complaints treated in the emergency room and valued the opportunity to work through clinical reasoning [30], and the real-time digital interaction simulates bedside teaching, which can increase exposure to a wider variety of patients and provide opportunities to practice clinical reasoning skills [31]. In fact, in our institution, the educational committee continuously implement mixed type teaching and assessments during the COVID-19 pandemic in the curriculum of medical clerkship. However, the result of our study suggested that these approaches are still difficult to completely replace direct primary care and approaching of patients during the clerkships. Therefore, educational committee recently announced that medical students should return to clerkship rotations and clinical trainings as soon as possible after getting vaccinated to gain their certification for knowledge and skills of universal precautions. Our medical students are also requested to learn how healthcare professionals collaborate in times of crisis, since they are the clinicians of the future [32].

Our study has some limitations. First, the present study was performed in a single teaching hospital in north Taiwan, where the pandemic was more severe than the rest of Taiwan since the number of infected people was the majority in north Taiwan. Therefore, the results may not be generalized to all the medical students and must be validated by further research in different hospitals from central, south, and even east Taiwan to obtain an overview of the impact of COVID-19 on medical students’ clinical learning outcomes. Second, the clinical competencies include several dimensions, such as medical knowledge, patient care, procedural and communication skills. The OSCEs may have limited value for evaluating some dimensions such as the system-based practice of the core competencies of medical students. Another limitation was that the present study is not a controlled research and there are some confounding factors. For example, though all medical students were assigned to internal medicine, surgical medicine, pediatrics, gynecology, and emergency medicine, the sub-specialties that everyone went to were not exactly the same, may cause different learning opportunities for every student.

## 5. Conclusions

In conclusion, our study shared the impact of the COVID-19 pandemic on the learning outcomes of clinical clerkship through serial OSCEs objectively. The degree of incremental improvement of performance between the regular and pandemic-impacted group of medical students on the standardized patient-based OSCE rather than skill-based OSCE stations suggested the importance of primary care in clerkship. Especially, more additional supplemental teachings are crucial in the emergency medicine course to ensure the completeness of the training of medical clerkship.

## Figures and Tables

**Table 1:** Descriptive statistics. * Results were considered significant by *p* < 0.05.

	Regular Group (*n* = 78)	Pandemic-Impacted Group (*n* = 80)	*p* Value
Gender (*n*, %)			
Male	52, 66.67%	52, 65%	0.700
Female	26, 33.33%	28, 35%	
Age (mean ± SD)	25.07 ± 0.99	25.14 ± 1.39	0.672
Number of stations with performance above standard (mean ± SD)			
First mock OSCE	4.35 ± 1.39	4.74 ± 1.23	0.062
Second mock OSCE	4.47 ± 1.32	4.69 ± 1.18	0.284
National OSCE	11.05 ± 1.34	10.38 ± 1.32	0.002 *
Total score of OSCEs (mean ± SD)			
First mock OSCE	747.07 ± 101.11	757.44 ± 95.21	0.508
Second mock OSCE	793.08 ± 83.25	796.83 ± 95.51	0.793
National OSCE	872.18 ± 66.50	834.96 ± 84.65	0.003 *

**Table 2:** Score increment between first mock OSCE and national OSCE.

	Regular Group			Pandemic-Impacted Group		
	Male (*n* = 52)	Female (*n* = 26)	*p* Value	Male (*n* = 52)	Female (*n* = 28)	*p* Value
Total score increment (mean ± SD)	126.55 ± 96.43	122.22 ± 94.59	0.851	93.02 ± 104.33	50.26 ± 97.83	0.075
Standardized patient-based station score increment (mean ± SD)	76.99 ± 72.72	73.91 ± 77.34	0.863	37.63 ± 86.45	6.53 ± 80.92	0.118
Skill-based stationscore increment (mean ± SD)	49.56 ± 50.04	48.31 ± 49.04	0.917	55.39 ± 44.60	43.73 ± 34.59	0.197

## Data Availability

Most of the information is already listed in the article, we will provide the original file by request.
